# The Innate and Adaptive Immune System as Targets for Biologic Therapies in Inflammatory Bowel Disease

**DOI:** 10.3390/ijms18102020

**Published:** 2017-09-21

**Authors:** Grainne Holleran, Loris Lopetuso, Valentina Petito, Cristina Graziani, Gianluca Ianiro, Deirdre McNamara, Antonio Gasbarrini, Franco Scaldaferri

**Affiliations:** 1 Internal Medicine, Gastroenterology and Liver Unit, Gastroenterology Area, Fondazione Policlinico Universitario Gemelli, Università Cattolica del Sacro Cuore, 00168 Rome, Italy; lopetusoloris@libero.it (L.L.); valepetito88@gmail.com (V.P.); graziani.cristina@gmail.com (C.G.); gianluca.ianiro@hotmail.it (G.I.); antonio.gasbarrini@unicatt.it (A.G.); francoscaldaferri@gmail.com (F.S.); 2 Gastroenterology Department, Department of Clinical Medicine, Trinity College Dublin, Dublin 2, Ireland; mcnamad@tcd.ie

**Keywords:** inflammatory bowel disease, innate immunity, adaptive immunity, molecular targets, biologic therapies, Anti-TNF, Anti-integrins, inflammatory cytokines

## Abstract

Inflammatory bowel disease (IBD) is an immune-mediated inflammatory condition causing inflammation of gastrointestinal and systemic cells, with an increasing prevalence worldwide. Many factors are known to trigger and maintain inflammation in IBD including the innate and adaptive immune systems, genetics, the gastrointestinal microbiome and several environmental factors. Our knowledge of the involvement of the immune system in the pathophysiology of IBD has advanced rapidly over the last two decades, leading to the development of several immune-targeted treatments with a biological source, known as biologic agents. The initial focus of these agents was directed against the pro-inflammatory cytokine tumor necrosis factor-α (TNF-α) leading to dramatic changes in the disease course for a proportion of patients with IBD. However, more recently, it has been shown that a significant proportion of patients do not respond to anti-TNF-α directed therapies, leading a shift to other inflammatory pathways and targets, including those of both the innate and adaptive immune systems, and targets linking both systems including anti-leukocyte trafficking agents-integrins and adhesion molecules. This review briefly describes the molecular basis of immune based gastrointestinal inflammation in IBD, and then describes how several current and future biologic agents work to manipulate these pathways, and their clinical success to date.

## 1. Introduction

Inflammatory Bowel disease (IBD) is a multifactorial chronic relapsing immune-mediated inflammatory condition composed of two main subtypes, namely Crohn’s disease (CD) and Ulcerative Colitis (UC). The incidence and prevalence of IBD is increasing worldwide. It is known to be highest in Western countries, specifically North America and Northern Europe, however there has also been a substantial increase in the presentation of IBD in developing countries, particularly Africa, and in Australasia where prevalence had previously been relatively low [1]. Several risk factors for the development of IBD have been identified including genetic factors, smoking, dietary factors and microbial pathogens, many of which have given further insight into the pathogenesis of IBD. Although the exact etiology of IBD is still being defined, it is thought to be due to a complex interaction between many factors, including defects in the immune system, both innate and adaptive; microbial dysbiosis, i.e., abnormal levels of, or abnormal response to, the gastrointestinal microbiome; a genetic predisposition; and a number of environmental factors [2]. At present, it is not fully understood which of these factors are the initiators of inflammation and which are compounders, however, as an autoimmune condition, there is accepted recognition of the role of the innate immune system to initiate inflammation, followed by the adaptive immune system to maintain this inflammation and cause progression of disease.

The medical treatment of IBD focuses on controlling active and chronic gastrointestinal inflammation and preventing disease progression to induce clinical, biochemical, endoscopic and histological remission. Generally, a combination of medical therapies is used depending on the type, severity and location of disease, including systemic and topical steroidal and 5-aminosalicylate compounds, and immunosuppressive agents such as Azathioprine, 6-mercaptopurine and methotrexate [3,4]. In more refractory cases, or in the management of severely active or fulminant disease, more potent immunosuppressive agents such as cyclosporine, calcineurin inhibitors and biologic agents are used. Additionally, clinical trials are currently underway evaluating the use of mesenchymal stem cell transplantation in IBD, and bone marrow transplantation has been used in rare cases [5,6]. The major focus of therapeutic development to date has been in targeting various aspects of the immune system, based on the identification of specific immune-mediated inflammatory targets identified in IBD. This has been most successfully achieved over the last decade or so via the use of biologic agents, which comprise proteins or other substances derived from a biological source. Of these, anti-tumor necrosis factor (TNF-α) focused treatments have been the most widely used, however levels of efficacy have been extremely variable with initial non-response rates of over 30%, followed by a secondary loss of response of 30% [7].Identifying immune treatment targets for IBD is a rapidly evolving area of research, however, the unpredictable variability in response rates suggests a very complex and still unidentified interaction between factors and an individual patient variability. In addition, in vivo and in vitro results are often not reproducible in real life clinical studies, which may be due to the important influence of the gut microbiome on gastrointestinal immunity. The recent introduction of more ambitious treatment targets including mucosal, histological and trans-mural healing calls for the development of more personalized biologic treatments. However, short and longer term safety concerns regarding the use of biologics including the risks of infectious, allergic, malignant and immune mediated complications need to be fully delineated before these newer medications can be incorporated into clinical practice.

**Figure 1:**
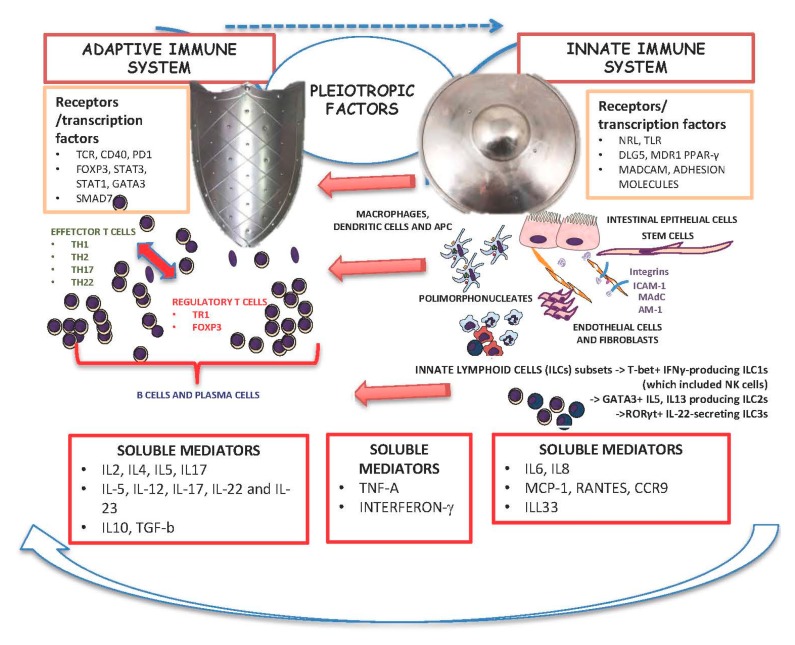
Innate and adaptive immune system.

**Figure 2:**
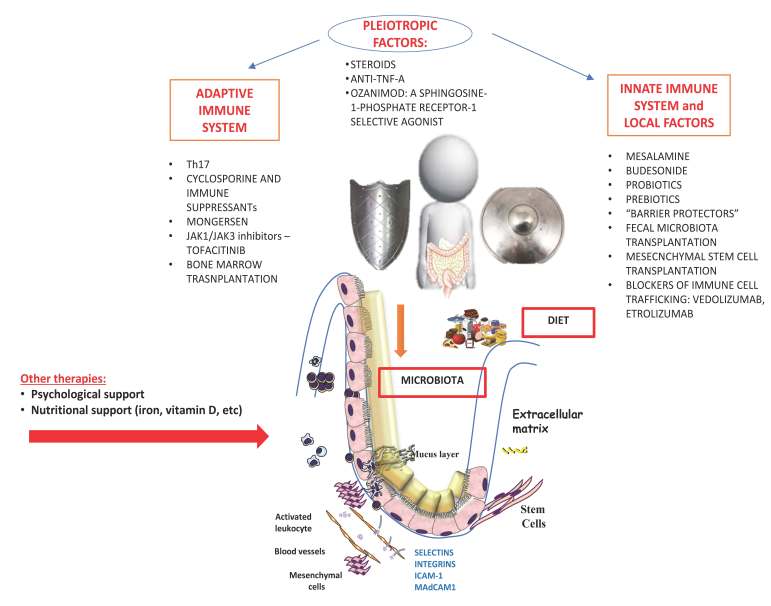
New drugs in IBD (inflammatory bowel disease) targeting innate and adaptive immune system.

This review firstly gives a basic overview of the role of the innate and adaptive immune systems in IBD pathogenesis (Figure 1), and secondly on the use of these factors as treatment targets by biological agents in IBD (Figure 2). We discuss currently available treatments and those in pre-clinical use.

## 2. Components of the Innate Immune System

In innate immunity, pathogens are recognized and responded to in an immediate non-specific way, and cells involved in this type of response include neutrophils and other types of granulocytes, macrophages, natural killer (NK) cells, innate lymphoid cells, and mast cells. Adaptive immunity depends upon the specific recognition of antigens by B or T cell receptors, and as a result this type of response is slower than innate immunity. Macrophages and dendritic cells (DCs), acting as antigen-presenting cells (APCs), link the innate and the adaptive immunity, due to their dual roles in both secreting cytokines which stimulate innate immune cells, in addition to presenting antigens to the T cells as major histocompatibility complex (MHC) molecules [8]. The innate immune system is comprised of the mucus and epithelial barrier, macrophages, monocytes, neutrophils, DCs, NK cells, eosinophils, and basophils [9]. A physical barrier of immunity is provided in the gut wall by the presence of a protective mucus layer generated by goblet cells, and the maintenance of a relatively impermeable epithelial barrier between the immune cells and the intraluminal microbiome [10]. Genome-wide association studies (GWAS) in IBD have demonstrated several susceptibility genes involved in innate mucosal defense and antigen presentation and have specifically identified *DLG5*, *MDR1*, *NOD2* and *PPAR-γ* genes as important players in this process [11,12]. These genetic defects, leading to deficiencies in mucus production and intestinal permeability, have been associated with an increased susceptibility to the development of IBD due to impaired pathogen recognition, reduced clearance of microbials and persistent antigenic stimulation with upregulation of cytokines [13,14,15]. Their detection has been helpful in uncovering some of the key molecular immune targets which have subsequently been developed as treatment targets, including several interleukins (ILs), tumor necrosis factor (TNF), nuclear factor-κB, and antisense oligonucleotides [16]. Beyond the barrier function of the innate immune system, intraluminal pathogens communicate with the innate immune system via several immune receptors including Toll like receptors (TLRs) and Nod like receptors (NLRs), which are important for developing tolerance to certain pathogens and promoting wound healing [17]. Following attachment to these receptors, the recognition of certain pathogen associated molecular patterns (PAMPs) by DCs and macrophages occurs, leading ultimately to the activation of several signaling pathways, and the production of pro-inflammatory cytokines, chemokines and antimicrobial peptides [18]. In addition, DCs and macrophages both have an important role in linking the innate and adaptive immune system, by acting as APCs. DCs work by migrating to peripheral sites of lymphoid tissue where they initiate specific T cell responses and attach a homing signal to leucocytes via certain integrins and selectins. DCs are thought to remain in a low level of activity and a ”tolerogenic state” in healthy mucosa, however in IBD dramatic changes have been shown in the levels of specific TLRs on DCs, with further significant differences noted between CD and UC [19]. Traditionally, macrophages can be categorized as either classically or alternatively activated by certain pathogens, leading to the secretion of various cytokines (TNF, IL-1, IL-10, IL-12, IL-18, and IL-23), chemokines and oxidative chemicals, and ultimately the regulation of either Th1 and Th17, or Th2 mediated immune responses, respectively, alongside a direct phagocytic function [20]. However, gut resident macrophages, particularly those of IBD patients, cannot be as easily categorized as those located elsewhere, as they have been shown to have higher rates of phagocytic activity and an increased secretion of cytotoxins [21]. In addition, the innate immune system uses autophagy to protect its integrity and maintain gut homeostasis via the secretion of bactericidal compounds such as antimicrobial peptides (AMPs), defensins and lysozyme, the cytotoxic activity of NK cells and the secretion of epithelium-protective transforming-growth factor (TGF-b) by T regulatory (Treg) cells of the mucosal lamina propria [22]. Over the past decade, there has been an increased recognition of the importance of innate lymphoid cells, previously called innate helper or natural helper cells, which have become a major target for treatment development. In contrast to DCs and macrophages, they do not express antigen-specific receptors, instead, their function is regulated by the cytokines released from APCs and other cells present in damaged or inflamed tissues [23]. They respond immediately to pathogenic stimuli by releasing further cytokines (including interferon (IFN-γ), IL-5, IL-12, IL-17, IL-22 and IL-23) and other mediators in a bid to prevent escalation of inflammation, however they have also been implicated in causing chronic intestinal cell inflammation [24,25,26]. The cytokines secreted by innate lymphoid cells are similar to those secreted by the T helper cells of the adaptive immune system. NK cells, which were previously thought to be the only innate immune cells of lymphoid origin, are now known to be a subtype of the innate lymphoid cell group.

## 3. Components of the Adaptive Immune System

One of the primary steps in the initiation of the adaptive immune response involves activation of the Th lymphocytes (Th1, Th2, Th17 and Th22 cells) and suppression of the activity of Treg cells [27]. This is coordinated by the migration of DCs to peripheral lymphoid areas to activate antigen-specific naive T lymphocytes [28]. These activated T cells then proliferate and become memory and effector T cells which enter the circulation to migrate towards the initial site of binding of the antigen to DCs, often directed by certain gut homing signals mediated by DCs [29]. The integration of Th cells into the intestinal lamina propria is then mediated by adhesion molecules, such as selectins and integrins, and their ligands which are present in the endothelial cells of blood and lymphatic vessels [30]. In relation to IBD immunity, key gut homing mediators are the α4β7 integrin expressed by lymphocytes colonizing gastrointestinal lymphoid tissue, and the mucosal addressin-cell adhesion molecule 1 (MAdCAM-1) present on the endothelium of intestinal venules [31,32].

CD was traditionally thought to be a Th1 mediated disease, while the activation of Th2 cells was more commonly associated with UC, however more recently a significant overlap between the two pathways has been recognized, along with the contributory role of Th9, Th17 and Th22 cells, and T regulatory (Treg) cells [33,34]. Th1 cells are thought to be activated predominantly by antigen presentation of microbial species, including bacteria, viruses and fungi, inducing IFN-γ and IL-12 release, which activates a transcription factor known as STAT1 (signal transducer and activator of transcription-1), further leading to the activation of transcription factor T-β [35]. The characteristic cytokines secreted by activated Th1 cells are INF-γ, IL-12 and TNF-α [36]. Th2 cell activation is predominantly induced by the secretion of IL-4, which leads to the activation of STAT6, and further activation of a transcription factor GATA-3 [37]. The characteristic cytokines released by activation of the Th2 cells are IL-4, IL-5, IL-13, IL-21 and IL-25. Induction of the Th17 pathway is thought to occur in the absence of IL-4 and IL-12, and the presence of IL-6, IL-23 and TGF-b, mediated by the activation of STAT3, and leads to the secretion of IL-17 and IL-22. Th22 requires IL-6 and TNF-α mediated activation of the STAT3 pathway, leading to the secretion of IL-22, IL-13, TNF-α, fibroblast growth factor (FGF), and chemokines. The role of Treg cells is to control chronic inflammation by suppressing the immune response by downregulating the expression of MHC molecules, and promoting tolerance to inflammatory stimulants [38]. The expression of transcription factor Fox3p is critical for the development and function of Treg cells and they are induced by high levels of TGF-β.

## 4. Interplay between the Innate and Adaptive Immune System in IBD and the Influence of Pleiotropic Factors as Targets for Current Treatment of IBD

The immunopathogenicity in IBD is thought to be due to the initial activation of the innate immune system causing a non-specific response, followed by the upregulation and maintenance of this inflammation by activation of the adaptive immune system, involving various feedback loops generating and sustaining chronic inflammation. The specific triggers of activation of the immune system in IBD are largely unknown and very complex but are thought to be immune and non-immune based, with an accepted role of the gut microbiota and non-immune derived cells of the inflammatory cascade including chemokines and inflammasomes [39]. We suggest a simplified approach to dissecting the mechanism of action of current therapies for IBD, as outlined in Figure 2 by considering: firstly, those acting on the adaptive immune system; secondly, those acting on the innate immune system and local luminal factors; and, thirdly, those acting on the pleiotropic factors, which influence both the innate and adaptive immune systems. Finally, we cannot overlook the essential basic components of health in IBD and the invaluable impact of adequate nutritional and psychological support.

## 5. Targeting the Adaptive Immune System

### 5.1. Inhibitor of Th1/Th17-Anti-Cytokine-IL 12/23

The pro-inflammatory cytokine IL-12 family, which includes IL-22, IL-23, IL-25 and IL-27, is responsible for the differentiation of T helper (Th) cells into Th1 cells and has been recently linked to the pathophysiology of CD as well as other immune-mediated disorders [40]. IL-12 and IL-23 are heterodimeric proteins composed of a unique subunit linked to a shared p40 subunit and studies have shown that patients with CD have an elevation of these subunits [41,42]. Ustekinumab is a fully human immunoglobulin G1κ monoclonal antibody against IL-12p40, thus inhibiting receptors for both IL-12 and IL-23 on T cells, NK cells, and APCs [43]. Ustekinemab is now FDA approved for the treatment of moderate-severe CD, and its efficacy was evaluated by the UNITI studies 1, 2 and 3, and reported by the UNITI working group [44,45]. Overall, these studies reported an initial clinical response rate of up to 34.3%, and remission rates of up to 53.1% in patients with moderate–severe CD with previous anti-TNF-α failure. Ustekinemab is administered via a weight-based intravenous infusion for induction, followed by a standard dose subcutaneous injection for maintenance. The UNIFI study (Clinical trials number NCT02407236) is currently underway to evaluate the use of Ustekinemab in UC.

Other monoclonal antibodies targeting the Th1/Th17 pathway using IL-23 as a target have been developed (including Briakinumab (an IL-12p50 blocker), MED12070 (an IL-23p19 blocker), and Risankizumab (a selective 1L-23 inhibitor), but have shown a lower efficacy than Ustekinumab, and none have reached phase 3 trials as a result [46,47,48].

### 5.2. Anti-SMAD7—Mongersen

TGF-β1 is an immunosuppressive cytokine with the ability to suppress pathogenic T cell activity and APC responses, which is an important agent in intestinal cell healing and the attenuation of inflammation [49]. Patients with IBD are known to have a deficiency in the activity of TGFβ1 which is thought to be due to increased levels of SMAD7, an intracellular protein that binds TGF-β1 receptor and prevents TGF-β1-associated and SMAD-associated signaling. Mongersen is an oral drug which downregulates SMAD7 activity after delivery predominantly to the terminal ileum and right colon, and is currently under evaluation for use in CD. Initial Phase 2 studies showed clinical response rates of up to 72%, with response being dose-dependent, however all doses showed a significantly higher efficacy than placebo [50]. Phase 3 studies are currently underway; however, it must be noted that, in its current form, i.e. a modified-release pH dependent tablet, the site of action is specifically limited to the terminal ileum and right colon, meaning that, in patients with more complex CD, or post-operative recurrence it may not be of value [51].

### 5.3. JAK1/JAK3 Inhibitors—Tofacitinib

Janus kinases are a family of intracellular non-receptor tyrosine protein kinases which play a key role in the pathogenesis of several autoimmune diseases [52]. Genome wide association studies have identified single nucleotide polymorphisms (SNPs) in the genome associated with many autoimmune conditions, including IBD, and have identified certain cytokines and receptor targets for treatment development via the JAK/STAT signaling pathway including IL-1, IL-6, IL-12, IL-23 and STAT3 [53]. A few orally administered small molecule inhibitors against various stages of the JAK/STAT pathway have been developed, with one of the therapeutic advantages of JAK inhibitors over monoclonal antibodies is that monoclonal antibodies are limited to block one specific molecule, while JAK inhibitors can target a wide range of down-stream signaling pathways induced by various inflammatory cytokines [54].

Tofacitinib is a small orally administered molecule which inhibits mainly JAK1 and JAK3, and to a lesser extent JAK2 and TYK2, affecting both the innate and adaptive immune responses by suppressing differentiation of TH 1 and 2 cells, with an additional effect on the pathogenic production of TH17 induced cytokine production [55]. Phase II and III studies on the use of Tofacitinib in both anti-TNF-α failure and naïve patients with moderate-severely active UC showed a dose dependent response with significantly higher rates of clinical remission compared to placebo [56,57]. Recent phase II trials on its use in moderate-severely active CD have been less promising, showing only a minor and statistically insignificant treatment effect compared to placebo, however a high rate of placebo response has been noted in all trials to date, and it is hypothesized that Tofacitinib may have a slower onset of action in CD compared to UC [58]. Despite its ability to target a wide range of inflammatory pathways, the side effect profile of Tofacitinib is reportedly similar to that of other monoclonal antibodies used in IBD, with no increased risk of infection or allergic reactions.

More recently, Vermiere et al. published results of the FITZROY study, a phase 2 randomized placebo controlled trial evaluating the use of a JAK1 selective inhibitor, Filgotinib, in patients with moderate-severely active CD [59]. They reported a significantly higher rate of clinical remission in the treatment group vs. placebo (47% vs. 23%, *p* = 0.008) after 10 weeks of treatment. Filgotinib has been shown to have a higher treatment efficacy than Tofacitinib in CD and phase III studies are currently underway evaluating its use in both CD and UC.

### 5.4. IL-13—Tralokinumab

IL-13 is a key cytokine involved in the Th2 immune response, and its upregulation has been proposed to be a main driver of mucosal inflammation in UC [60,61]. Mouse studies identified an upregulation of IL-13 in colitis, which could be prevented by blocking the interaction of IL-13 with its signaling receptor [62]. Tralokinumab is an IL-13 specific human immunoglobulin G4 monoclonal antibody that binds to and neutralizes IL-13, which showed therapeutic advantage in clinical trials involving patients with asthma and idiopathic pulmonary fibrosis. The results of a phase IIa clinical trial of the use of subcutaneous Tralokinumab for 12 weeks as an add-on therapy in patients with moderate-severely active UC unresponsive to other treatments were published in 2015 [63]. This study showed no significant differences in clinical response rates compared to placebo (38% vs. 33% *p* = 0.406), however there were higher rates of clinical remission (18% vs. 6% *p* = 0.033) and a trend towards higher rates of mucosal healing (32% vs. 20% *p* = 0.104) in the treatment group, suggesting some therapeutic benefit which warrant further clinical trials in selected patient groups.

## 6. Targeting Pleiotropic Factors between the Adaptive and Innate Immune Systems

### 6.1. Anti-TNF: Infliximab, Adalimumab, Golimumab, and Certolizumab

TNF-α is a pro-inflammatory cytokine produced mainly by activated macrophages, monocytes and T lymphocytes, and is chronically elevated both in intestinal cells and systemically in IBD patients [64,65]. TNF-α contributes to mucosal inflammation through several different mechanisms including: destruction of the intestinal barrier, induction of apoptosis of epithelial cells, secretion and induction of other cytokines including IL-1, IL6, adhesion molecules, leukocytes and metalloproteinases [66,67]. Biologic agents used against TNF-α are known as anti-TNF agents. There are several anti-TNF monoclonal antibodies currently approved for use in IBD including Infliximab, Adalimumab, Golimumab and Certolizumab. The reduction in gastrointestinal cell inflammation due to binding of TNF-α and neutralization of its activity works in several ways and is mainly mediated through the prevention of TNF-α receptor activation. TNF-α blockage has been shown to result in reduced intestinal permeability through decreased endothelial cell apoptosis and decreased permeability of tight junctions, increased Treg cell activity, reduced activity of various inflammatory mediators and T cells, and a reduction in inflammation mediated mucosal angiogenesis by preventing the production of vascular endothelial growth factor A from intestinal fibroblasts [68,69,70,71].

Infliximab, the first anti-TNF-α antibody to be FDA (Food and Drugs administration) approved, is a chimeric monoclonal antibody directed against TNF-α, composed of a variable region of murine origin comprising 25%, and a constant region of human origin comprising the remaining 75%, joined through disulfide bonds. Infliximab binds to both soluble and transmembrane forms of TNF-α with high affinity [72]. It is administered via intravenous infusions at a weight-based dose, and is approved for both induction and maintenance of remission in both CD and UC as published in both the ACCENT I and ACT I trials [73,74]. These trials showed initial clinical response rates using standard dosing regimens of up to 58% and 65%, and remission rates of up to 45% and 49% for CD and UC respectively. More recently, however, it has been shown that a third of patients are initial non-responders to Infliximab, with a further third developing secondary loss of response within a year of commencing treatment despite the use of therapeutic drug monitoring (the assessment of drug and TNF-α levels and anti-drug antibodies) to guide dose changes and addition of immunosuppressant therapy [75,76].

Adalimumab is a fully humanized anti-TNF-α antibody administered via subcutaneous injection, approved for both induction and maintenance of remission in both CD and UC. Its efficacy was initially reported by the CLASSIC I and CHARM studies for CD, and the ULTRA 1, 2 and 3 trials for UC showing initial clinical response rates of up to 36% and 55%, and remission rates of up to 36% and 30%, for CD and UC, respectively [77,78,79].

Golimumab is a fully humanized monoclonal IgG1 antibody against TNF-α that differs from Adalimumab by having an amino acid sequence identical to that of Infliximab [80]. It is approved for the treatment of moderate-severely active UC, and its clinical efficacy was evaluated by the PURSUIT I and II trials which showed an initial response rate of up to 55%, and a remission rate of up to 50% [81]. Golimumab is administered via subcutaneous injection, after efficacy trials demonstrated that drug levels were higher using the subcutaneous route rather than the intravenous route [82].

Certolizumab pegol is a 95% humanized pegylated Fab fragment of a monoclonal IgG anti-TNF-α antibody which does not contain an Fc portion, unlike the other available anti-TNF-α antibodies and has been shown not to demonstrate Fc mediated cellular cytotoxicity [83,84]. Certolizumab pegol is administered via subcutaneous injection and is licensed for the treatment of moderate-severe CD, including patients who have failed treatment with other anti-TNF-α agents [85]. Its efficacy was assessed by the PRECISE trials 1 and 2, and 3 which showed initial response rates of up 37%, remission rates of up to 48%, with sustained of remission of 76% at seven years in responders, and 13% of initial participants overall [86,87,88].

Not all anti-TNF-α agents have demonstrated therapeutic efficacy in IBD, with Etanercept a genetically engineered fusion protein fused with the Fc domain of human IgG1 demonstrating no benefit over placebo [89]. A study by Perrier et al. demonstrated that the binding to and blockage of the transmembrane form of TNF-α signaling is central to the efficacy of anti-TNF-α antibodies, which explains the inefficacy of Etanercept, as it neutralizes only the soluble form of TNF-α and is unable to block the transmembrane form [90]. A significant area of research in anti-TNF-α agents in IBD at present is the mechanism behind primary and secondary non-response. Initial importance was placed on decreased drug trough levels and the development of anti-drug antibodies leading to increased clearance of the drug, however, there have also been reports of higher levels of certain MMPs in non-responders, leading to drug cleavage and inactivation, and finally the suggestion of non-TNF-α mediated inflammation in certain IBD sub-groups, or the inability of anti-TNF-α agents to reach the level of intestinal inflammation [91,92,93].

### 6.2. Ozanimod: Yes a Sphingosine-1-Phosphate Receptor-1 Selective Agonist

The sphingosine-1-phosphate receptor-1 (S1P_1_) not required agonist ozanimod is a novel oral agent that has been shown in phase 2 clinical trials to be efficacious in treating UC, and trials of its use in CD are underway [94]. S1P_1_ is expressed by lymphocytes, dendritic and endothelial cells, and mesenteric lymph nodes, and, prior to a recent study by Karuppuchamy et al., its specific method of action in IBD had not been fully delineated [95]. This study determined three specific modes of action of S1P_1_ in IBD. The first targets lymphocytes in their earliest phase of trafficking by causing the retention of naïve T cells in secondary organs, secondly by causing the mobilization of effector T cells from the intestine and activated DCs to specific areas of action, and thirdly via the modification of the intestinal barrier via downregulation of the endothelial integrin ligands including MAdCAM-1 and VCAM-1, thereby tightening the intestinal barrier. The resultant net effect of action of this drug has been to reduce intestinal inflammation. In UC it has been shown to induce clinical remission in over 50% of patients, which is significantly higher than the placebo group. As with other leukocyte trafficking based drugs, the efficacy is thought to improve with a longer duration of treatment and results from Phase 3 trials in UC and phase 2 trials in CD are currently awaited.

### 6.3. Anti-IL-6: Tocilizumab Yes a Colon Is More Appropriate

IL-6 is predominantly produced by cells of the innate immune system including mast cells, macrophages and neutrophils [96]. The presence of IL 6 has been shown to be an essential component for activation of various pathways in the adaptive immune system including Th17 and Th2 responses [97]. It has been shown that in actively inflamed mucosa in IBD tissue and serum levels of IL-6 are elevated and correlate with activity of disease [98,99]. An anti-apoptotic role of IL-6 in IBD has been demonstrated due to the activation of STAT 3 and the induction of anti-apoptotic genes such as bcl-2 and bcl-xl within mucosal T cells. Tocilizumab, a recombinant humanized monoclonal antibody of the IgG1 subclass directed against soluble and membrane bound IL-6 is currently licensed for use in several rheumatic conditions and has been used successfully in pilot studies and case reports in IBD patients, however concerns have been raised regarding a higher rate of intestinal perforation associated with the use of Tocilizumab [100,101,102]. A few antibodies in pre-clinical trials have been developed against IL-6 including sirukumab and olokizumab, however no clinical trials in IBD have been undertaken [103,104].

### 6.4. Other Non-Biologic Therapies with a Pleiotropic Effect

The interface between the adaptive and innate immune system is the target of a number of therapies in IBD including systemic steroids, immunosuppressant medications and bone marrow transplantation.

## 7. Targeting the Innate Immune System and Local Factors

### 7.1. Leukocyte Trafficking: Anti-Integrins and Adhesion Molecules

Leukocyte trafficking refers to the process of attraction and gut-homing of leukocytes from the systemic circulation towards gastrointestinal endothelial cells, and subsequent adhesion to inflamed cells. Activated leukocytes present in gastrointestinal cells during inflammation lead to the release of multiple pro-inflammatory cytokines. In turn, these cytokines then upregulate the expression of certain adhesion molecules present on endothelial cells, including MAdCAM-1, ICAM-1 and VCAM-1 [32,33]. Integrins are heterodimeric receptors composed of α and β subunits and play a critical role in the adhesion cascade [105]. The major relevant components in IBD are α4β7 and α4β1.

### 7.2. Vedolizumab

Vedolizumab is an IgG1 humanized monoclonal antibody to the α4β7 integrin. Vedolizumab inhibits the adhesion of a subset of gut-homing lymphocytes to MAdCAM-1 which selectively downregulates gastrointestinal inflammation while preserving the systemic immune function [106]. Vedolizumab is administered via a standard dose intravenous infusion, and is approved for the treatment of moderate-severely active UC and CD. The efficacy for its use in UC and CD was evaluated in the GEMINI studies I and II which showed initial clinical response rates of up to 47.1% and 14.5% and remission rates at 52 weeks of 44.8% and 39% for UC and CD, respectively [107,108]. The GEMINI III study evaluated the use of Vedolizumab in patients with moderate-severely active CD who had previously failed treatment with at least one anti-TNF-α treatment and determined that Vedolizumab was not significantly more effective than placebo at inducing remission by Week 6; however, by Week 10, a significantly detectable efficacy over placebo was observed [109]. More recent longer-term data from the GEMINI LTS study have shown a sustained clinical response in in initial responders of up to 96% and 89% of patients with UC and C,D respectively, at 152-week follow up [110,111]. Both studies showed improved response rates after reduction of dosing interval in patients who had initially not responded to Vedolizumab. A recent study evaluating the long-term safety of Vedolizumab reported a low risk of serious infections or infusion reactions and no incidences of progressive multifocal leukoencephalopathy (PML) in over four years of its use, thought to be due to the gut selective properties of the medication [112]. Natulizumab, the first anti-integrin medication against both α4β7 and α4β1, used in IBD, was withdrawn from use for this indication in 2005 after three reported cases of PML associated with the medication [113].

### 7.3. Etrolizumab

Etrolizumab is a humanized IgG1 monoclonal antibody, which has a two-pronged therapeutic effect via the binding to the β7 subunit of α4β7 and the interaction between α_E_β_7_ and E-cadherin at the mucosal level [114]. Etrolizumab may represent an extremely valuable mechanism of action for the management of inflammation in IBD, mainly due to the concomitant blockade of α_E_β_7_-E-cadherin interaction which avoids the adhesion of intraepithelial T cells the epithelial cells. This makes Etrolizumab a particularly gut-selective therapy, as only 1–2% of circulating lymphocytes express α_E_β_7_, whereas it is present in over 90% of intraepithelial lymphocytes [115]. Phase II clinical trials have shown significant efficacy of Etrolizumab over placebo with initial remission rates of up to 21% in anti-TNF-α non-responders with moderate-severely active UC [116]. Phase III trials are currently underway to evaluate the long-term efficacy and safety of Etrolizumab in UC and CD.

### 7.4. Alicaforsen

Alicaforsen is a human ICAM-1 antisense oligonucleotide which blocks ICAM-1 production by complementary hybridization to the messenger ribonucleic acid (mRNA) of the target gene, therefore blocking protein translation [117]. Its use as an intravenous infusion in patients with CD showed inefficacy, as did trials of its use in UC when as a topical enema form. However, more recently a study by Patel et al. identified elevated serum levels of ICAM-1 in patients with pouchitis following panproctocolectomy for UC, suggesting that ICAM-1 might be a key player in driving the inflammation in pouchitis [118]. Studies using Alicaforsen in pouchitis have demonstrated significant clinical efficacy of up to 85% following a six-week course of treatment, however relapse occurred in over 80%, suggested prolonged treatment may be required [119].

### 7.5. Anti-MAdCAM-1 Antibody

The results of the TURANDOT study describing the use of a humanized IgG2K monoclonal antibody, PF-00547659, directed against MAdCAM-1 in UC was presented in abstract form at Digestive Diseases Week in 2015. By preventing the adhesion of the a4b7 integrin expressing calls to MAdCAM-1 clinical response was found in 54.2% and mucosal healing was found in 27.8%, which were both significantly higher than that in placebo treated patients [120]. However, there was no significant benefit over placebo when the same antibody was used in CD [121]. However, this study did demonstrate a better response in patients with a higher baseline CRP (C reactive protein) level, and previous reports of a longer required duration of treatment for all anti-adhesion based medications in CD over UC, further studies with a longer course of treatment are warranted to determine the actual treatment effect.

### 7.6. Manipulation of the Intestinal Microbiome: Fecal Microbiota Transplantation and Bacteriotherapy

The intestinal microbiome has been recognized as a key component of IBD pathogenesis, thought to work mainly by manipulation and activation of the innate immune system. A stable intestinal microbiota offers resistance against pathogenic bacteria, however; when the intestinal microbiota composition is disturbed, it allows a decrease in bacterial diversity and colonization resistance, allowing pathogenic bacteria to expand and cause disease. Animal models in IBD have provided two possible mechanisms of disease pathogenesis by the intestinal microbiota in IBD; either as an abnormal immune response to normal microbiota or a normal immune response to abnormal microbiota [122]. The process is thought to begin when there is an initial impairment in mucosal tolerance with a resultant coordinated response and activity of intestinal epithelial cells, goblet cells and paneth cells to preserve intestinal barrier function [123]. This can occur via changes in the mucus layer causing increased permeability occurring through several factors mediated by direct damage from secretions from bacteria or via immune-modulation of defensins and plasma cells [124,125]. Therapeutic manipulation of the intestinal microbiota in IBD has been assessed by two main routes: fecal microbiota transplantation (FMT) and the use of probiotics. Although perhaps not thought of traditionally as a biologic agent, there is still a lack of consensus regarding the categorization and medical approval of FMT and it has been recommended by the FDA to be considered a biologic drug [126].

FMT refers to the transfer of fecal material containing bacteria from a healthy individual to a diseased patient, with a view to correcting the underlying microbial dysbiosis. Multiple studies have shown the success and safety of FMT in the treatment of recurrent *Clostridium difficile* (*C. difficile*) infection and it is currently recommended in the treatment guidelines for antibiotic resistant *C. difficile* [127]. As the dysbiosis occurring in *C. difficile* infection and IBD is thought to be similar, this has formed the basis for its use in IBD. Multiple cohort studies have explored the efficacy of FMT in IBD with initial results indicating safety and efficacy, with clinical response and remission rates as high as 60% and 40% respectively [128,129,130,131]. The role of FMT in the treatment for IBD was examined by the expert panel of the European consensus conference on FMT in clinical practice and although deemed to be a promising area of research, it was felt that the heterogeneity and small numbers to date did not carry enough evidence to recommend its use in IBD at this point [132]. However, many randomized controlled clinical trials are currently underway which may influence future guidelines.

Probiotics are live microorganisms that when ingested can provide a beneficial effect on the host. There are difficulties in assessing their health benefits overall due to a wide heterogeneity in the commercially available products worldwide, however two main probiotics have been studied extensively in IBD: ECN (*Escherichia coli* Nissile) and VSL#3 (a mixture of eight different bacteria, four strains of Lactobacilli, three strains of Bifidobacteria, and one strain of Streptococcus). They work via several mechanisms including competitive inhibition of other microbials and production of short chain fatty acids, with a resultant increase in barrier function, improvements in the mucus layer and defense system, and activation of an anti-inflammatory effect [133]. Analysis of the efficacy of probiotics in CD has shown disappointing results to date, and their use is not currently advocated by any guidelines, however there have been more positive results for UC and pouchitis [134,135,136]. The strongest indication for probiotics has been in UC where ECN has been proven to be as effective as 5-aminosalicylates (5-ASAs) at maintaining remission, and is now recommended by European Crohn’s and Colitis Organisation (ECCO) as an alternative treatment to 5-ASAs [137,138]. VSL#3 has also been shown to be useful in both inducing and maintaining remission in pouchitis and active UC [139,140].

### 7.7. TLR Agonists and Antagonists

TLRS are important components of the innate immune system, expressed on APCs including macrophages, dendritic cells, T lymphocytes, and intestinal epithelial cells, and can be considered as an interface between the intestinal epithelial barrier, microbiota, and the immune system [141]. TLR activation is thought to occur in IBD due to microbial dysbiosis causing TLRs to recognize microbial DNA as pathogenic, thereby leading to the induction of several intracellular signaling cascades. This results in the production of cytokines and chemokines and the activation of transcription factor NF-κB, mitogen-activated protein (MAP) kinases and interferon regulatory factor 3. Significant research is underway to identify specific TLRs implicated in IBD pathogenesis that may become future therapeutic targets, and preclinical trials have begun to examine the therapeutic use of a few TLRs in IBD including OPN-401, a humanized IgG4 monoclonal antibody against TLR-2; and 1A6, A TLR-4 antagonist [142].

### 7.8. Kappaproct: TLR-9 Agonist

Activation of intracellular TLR9 drives the production of numerous pro-inflammatory cytokines, including TNF, IL-6, and IL-12, leading to a strong induction of the Th1-immune response [143]. Animal studies have shown that TLR9 is effective at reducing gastrointestinal epithelial cell apoptosis and that a deficiency in TLR 9 leads to an accumulation of Tregs and the inability to fight off infection [144]. Several experimental studies have demonstrated the ability of TLR9 to prevent intestinal cell inflammation and promote mucosal healing [145,146]. The COLLECT study by Atreya et al. describes the use of DIMSO150, a topical preparation of the TLR-9 agonist DNA-based immunomodulatory sequence 0150, given as two single doses four weeks apart, to 131 patients with refractory UC [147]. This study found that a significantly higher proportion of patients achieved clinical remission and mucosal healing at Week 4, however these findings were no longer statistically significant by Week 12. Phase II trials are currently underway to further investigate its role in UC.

### 7.9. Potential Emerging Target: IL-33/ST2 Axis

IL-33 is a member of the IL-1 family which signals via the ST2 receptor and in the presence of IL-33, ST2 pairs with its co-receptor, IL-1RAcP, and signals through mitogen-activated protein kinase (MAPK)- and NF-κB-dependent pathways. IL-33 is a recognized inducer of immune responses with a dual function, via its originally proposed action on Th2 cells and mast cells, and more recently via its action on innate lymphoid cells, Tregs, Th1 cells and NK cells [148,149]. Recent evidence, mainly in animal studies, has shown a significant role for IL-33 in epithelial restoration and repair in IBD, which have shown that the role of IL-33 is dependent on the stage of inflammation and the immunological status of the individual–being detrimental in the acute phase and beneficial in the recovery stage [150,151,152]. Multiple studies have shown increased tissue levels of IL-33 and the ST2 receptor in patients with IBD, particularly UC, compared to controls, and additionally elevated serum levels of IL-33 in UC patients were shown to be significantly decreased following treatment with anti-TNF treatment [153,154]. The precise role of both IL-33 and ST2 in the pathogenesis of IBD is still being delineated and several animal studies have been conducted to examine the role of therapeutic blockade of the IL-33/ST2 axis in IBD [155,156]. No clinical trials have been developed targeting this pathway specifically in IBD yet, however, due to its potential to prevent and reverse intestinal fibrosis, it represents and exciting area of treatment development [157].

### 7.10. Conventional Therapies Affecting the Innate Immune System and Local Factors

The target of action of both oral and local 5-aminosalicylates and steroids is on the innate immune system and locally on colonic mucosal cells.

## 8. Immunogenicity

A problem increasingly encountered with the more widespread use of biologic agents is that of immunogenicity. Immunogenicity refers to the propensity of biologic drug proteins to elicit an immune reaction against themselves in the recipient, leading to the development of anti-drug antibodies (ADAs), and it is known to be an important cause of non-response to biologic treatment. Certain biologic agents are known to be more immunogenic than others, with chimeric agents having a higher level of immunogenicity than fully humanized agents, in addition to several other pharmacodynamics properties. Furthermore, several prescribing factors have been shown to affect immunogenicity including the scheduling of dosing and the use of concomitant immunosuppressants. Episodic treatment or drug breaks have been associated with significantly higher levels of ADAs in many agents, and the use of immunosuppressants such as Azathioprine and Methotrexate is known to reduce ADA formation [158]. All biologic agents have been shown to promote development of ADAs to some extent, although the direct comparison of the significance of immunogenicity for different biologic agents have not been sufficiently undertaken due to differences in methodology, including variable sampling intervals and assay techniques [159]. Therapeutic drug monitoring (TDM), using both drug and ADA levels to guide titration of dosing of biologic agents has been suggested to improve interpretation of these factors, and may even be useful for predicting response to other biologic agents [160,161]. There have been many publications regarding the clinical utility of TDM in anti-TNF-α agents; however, there is limited knowledge available on the significance of TDM in newer agents including Vedolizumab and Ustekinemab, although several trials are underway to resolve this issue [162]. However, despite being an active area of research in IBD at present, doubts remain about the clinical significance of the presence of ADAs, and how to interpret their presence, and the consensus on their use is still being debated [163]. Further studies and consensus opinions regarding the use of TDM of biologic agents may help to increase the cost-effectiveness of their use and decrease rates of loss of response.

## 9. Biosimilars

Although biologic agents have clearly demonstrated clinical efficacy in the management of IBD, and potentially provide some economic advantage in terms of reducing the need for hospitalization and in some cases the need for surgical intervention, they remain an extremely expensive medication, and with treatment targets for IBD becoming more ambitious, it is likely that our dependence on biologic agents will increase going forward. Biosimilars are defined by the FDA as a biological product that is highly similar to a reference medicinal product, with no clinically meaningful differences in terms of safety, purity, and potency [164]. Due to the difficulty in replicating exact copies of biologic agents, biosimilars may not be identical to the reference product but the active ingredients remain the same and the FDA allows only subtle differences in the clinically active components included. Once the original patency on a biologic agent has expired, pharmaceutical companies are entitled to apply for FDA approval for biosimilar agents. Although it is hoped that this will introduce competition between pharmaceutical companies to reduce prices similar to other generic medications, there have been doubts raised about the direct comparison of biosimilars to the reference medication, and serious concerns regarding a potentially expedited FDA approval of biosimilars for all indications in which the reference medication is approved for, without a similarly comprehensive safety review. Biosimilars have been available in the European Union since 2006, with predictions of cost-saving across Europe of up to 30% for all inflammatory diseases [165]. An anti-TNF-α biosimilar, CT-P13 (marketed as Remsima and Inflectra), was the first biosimilar agent introduced for IBD in Europe in 2013, and is approved for the treatment of both UC and CD [166]. To date, the uptake of these agents has been limited by scarce availability of data regarding the safety, efficacy and immunogenicity of these agents in IBD. The largest study to date has been a Norwegian study describing the switching of 482 patients with chronic inflammatory diseases (including IBD, Psoriasis, Rheumatoid arthritis) from Infliximab to CT-P13, and no significant differences between the two medications in terms of disease worsening or antibody development, however the study was not sufficiently powered to detect differences based on specific disease subtype [167]. In addition, a recently published study describing the initial results of a managed switching program from Infliximab in the United Kingdom reported no significant differences between the two agents in terms of clinical efficacy and side effect profile [168]. Further longer term studies are required to compare the efficacy and particularly to assess immunogenicity of biosimilars in IBD but based on the available literature the ECCO have approved both the initiation of CT-P13 and switching to CT-P13 from Infliximab for both forms of IBD [169].

## 10. Conclusions

Biologic agents currently represent the most effective therapeutic agents at controlling and preventing the immune-mediated inflammation in IBD. Although our knowledge of the involvement of the immune system in the pathophysiology of IBD is still evolving, significant advances over the last two decades have led to the refinement of these biologic agents to have a more gut-specific effect and a more limited effect on systemic immunity overall, making them a likely safer treatment in the longer term. This review gives an overview of the currently available biologic agents for IBD, and their treatment targets within the immune system and hopefully gives the reader an understanding of the development of these medications to target more specific molecules in more recent times, with a view to improving efficacy and reducing adverse events. It is important to highlight how the clinical efficacy of these agents can be unpredictable, and often varies substantially from that reported in animal studies, both reflecting the individual variability in inflammatory pathways in each patient and the crucial role of the microbiota in shaping our immune response. However, the rapidly increasing availability of targets directed against many different inflammatory pathways means that our ability to provide personalized treatment to patients with IBD is becoming even more imminent.

## Abbreviations

IBD	Inflammatory Bowel Disease
CD	Crohn’s Disease
UC	Ulcerative Colitis
TNF	Tumor Necrosis Factor
APC	Antigen Presenting Cells
TLR	Toll-like Receptors
NLR	Nod-like Receptors
DC	Dendritic Cells
Th	T helper
Treg	T regulatory
IL	Interleukin
IFN	Interferon
NK	Natural Killer
TGF	Transforming Growth Factor
ECCO	European Crohn’s and Colitis Organization
FDA	Food and Drug Association
TDM	Therapeutic Drug Monitoring
ADA	Anti-Drug Antibodies
